# Wide QRS complex and the risk of major arrhythmic events in Brugada syndrome patients: A systematic review and meta‐analysis

**DOI:** 10.1002/joa3.12290

**Published:** 2019-12-27

**Authors:** Pattara Rattanawong, Jakrin Kewcharoen, Chol Techorueangwiwat, Chanavuth Kanitsoraphan, Raktham Mekritthikrai, Narut Prasitlumkum, Prapaipan Puttapiban, Poemlarp Mekraksakit, Wasawat Vutthikraivit, Dan Sorajja

**Affiliations:** ^1^ Department of Cardiovascular Medicine Mayo Clinic Phoenix AZ USA; ^2^ Faculty of Medicine Ramathibodi Hospital Mahidol University Bangkok Thailand; ^3^ University of Hawaii Internal Medicine Residency Program Honolulu HI USA; ^4^ Department of Medicine Einstein Medical Center Philadelphia PA USA; ^5^ Department of Internal Medicine Texas Tech University Health Sciences Center Lubbock TX USA

**Keywords:** Brugada syndrome, Major arrhythmic events, wide QRS

## Abstract

**Background:**

Brugada syndrome (BrS) is an inherited arrhythmic disease associated with an increased risk of major arrhythmic events (MAE). Previous studies reported that a wide QRS complex may be useful as a predictor of MAE in BrS patients. We aimed to assess the correlation of wide QRS complex with MAE by a systematic review and meta‐analysis.

**Methods:**

We comprehensively searched the databases of MEDLINE and EMBASE from inception to June 2019. Included studies were cohort and case control studies that reported QRS duration and the relationship between wide QRS complex (>120 milliseconds) and MAE (sudden cardiac death, sudden cardiac arrest, ventricular fibrillation, sustained ventricular tachycardia, or appropriate shock). Data from each study were combined using the random‐effects model.

**Results:**

Twenty‐two studies from 2007 to 2018 were included in this meta‐analysis involving 4,814 BrS patients. The mean age was 46.1 ± 12.8 years. The patients were predominately men (77.6%). Wide QRS duration was an independent predictor of MAE (pooled risk ratio 1.55, 95% confidence interval: 1.04‐2.30, *P* = .30, *I* ^2^ = 38.4%). QRS duration was wider in BrS who had history of MAE (weight mean difference = 8.12 milliseconds, 95% confidence interval: 5.75‐10.51 milliseconds).

**Conclusions:**

Our study demonstrated that QRS duration is wider in BrS who had history of MAE, and a wide QRS complex is associated with 1.55 times higher risk of MAE in BrS populations. Wide QRS complex can be considered for risk stratification in prediction of MAE in patients with BrS, especially when considering implantable cardioverter‐defibrillator placement in asymptomatic patients.

## Graphical abstract

Our study demonstrated that a wide QRS complex is associated with 1.55 times higher risk of major arrhythmic events in Brugada syndrome populations. Wide QRS complex can be considered for risk stratification in prediction of MAE in patients with BrS, especially when considering implantable cardioverter‐defibrillator placement in asymptomatic patients.

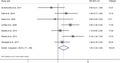

**Abbreviations**
BrS: Brugada syndrome
CI: confidence interval
ECG: electrocardiogram
MAE: major arrhythmic events
NOS: Newcastle‐Ottawa quality assessment scale
RR: risk ratio
SCA: sudden cardiac arrest
SCD: sudden cardiac death
VF: ventricular fibrillation
VT: ventricular tachycardia

## 1 INTRODUCTION

Brugada syndrome (BrS) is an inherited arrhythmic disease associated with an increased risk of major arrhythmic events (MAE) and sudden cardiac death (SCD), and ventricular arrhythmias such as ventricular fibrillation (VF) and ventricular tachycardia (VT). It is characterized by a coved‐type ST elevation and atypical right bundle‐branch block appearances in the right precordial leads. The disease burden of BrS is difficult to determine; however, data suggest that the prevalence of asymptomatic patients with Brugada ECG pattern varies among different populations, ranging between 0% and 0.4%.^1^, ^2^, ^3^ With SCD being the most worrisome manifestation of BrS, identifying patients who would benefit from an implantable cardioverter‐defibrillator for primary prevention is critical; however, ventricular arrhythmia risk stratification remains challenging and controversial.

Many electrocardiogram (ECG) abnormalities have been proposed as markers for risk of MAE. Among them, conduction disturbances, such as delayed depolarization manifested as a wide QRS complex, may contribute to the development of MAE in BrS by providing or worsening a pro‐arrhythmic substrate.^4^ Previous studies reported that QRS duration may be useful as a predictor of MAE in BrS patients. Wide QRS complex, as defined by QRS duration >120 milliseconds measured on a standard 12‐lead ECG, has been associated with an increased risk of ventricular arrhythmia.^5^ Additionally, wide QRS complex was also found to be more prominent in symptomatic BrS patients.^6^, ^7^ However, data from other studies reported conflicting results, suggesting that QRS width was not useful as a risk stratification tool.^8^, ^9^ Therefore, we aimed to assess whether wide QRS complex is associated with an increased risk of MAE in BrS patients by performing a systematic review and meta‐analysis.

## 2 METHOD

### 2.1 Search strategy

Two investigators (CK and NP) independently searched for published studies indexed in MEDLINE and EMBASE databases from inception to June 2019 using a search strategy that including the terms “ECG”, “QRS”, and “Brugada” as described in File S1. Only full articles in English and studies conducted in cohorts were included. A manual search for additional pertinent studies and review articles using references from retrieved articles was also completed.

### 2.2 Inclusion criteria

The eligibility criteria included the following:

1. Cohort (prospective or retrospective), cross‐sectional, or randomized control trial studies reporting follow‐up outcome of MAE including SCD, sudden cardiac arrest (SCA), VF, sustained VT and appropriate shock in BrS patients with and without previously documented wide QRS complex as well as history of MAE as a characteristic.
2. QRS duration, adjusted or unadjusted risk ratio (RR), odds ratio, hazard ratio with 95% confidence interval (CI), or sufficient raw data for the calculation were provided. Patients without previously documented wide QRS complex were used as controls. Odds ratio and hazard ratio were converted to RR by previously reported principal equations.^10^

Study eligibility was independently determined by two investigators (RM and CT) and differences were resolved by mutual consensus. The Newcastle‐Ottawa quality assessment scale (NOS) was used to assess each study's quality in three domains, recruitment and selection of the participants, similarity and comparability between the groups, and ascertainment of the outcome of interest among cohort and case‐control studies.^11^

### 2.3 Data extraction

A standardized data collection form was used to obtain the following information from each study: title of study, name of first author, year of publication, study design, country of origin, number, gender and age of the participants, Brugada ECG pattern, available MAE outcome, follow‐up duration, leads of QRS duration measurement, and confounders that were adjusted in the multivariable analysis, if available.

Two investigators (PP and PM) independently performed this data extraction process to ensure accurate data extraction. Any data discrepancy was resolved by referring back to the original articles.

### 2.4 Definition

#### 2.4.1 Wide QRS complex

Wide QRS complex was defined as QRS complex with a duration of >120 milliseconds.

#### 2.4.2 Brugada syndrome

Brugada syndrome was diagnosed in patients with ST‐segment elevation with type 1 morphology ≥2 mm in ≥1 lead in the right precordial leads V1, V2, positioned in the 2nd, 3rd, or 4th intercostal space occurring either spontaneously or after provocative drug test with intravenous administration of class I antiarrhythmic drugs.^12^

#### 2.4.3 Major arrhythmic event

Major arrhythmic events were defined by either of SCD, SCA, VF, sustained VT, or appropriate shock. VF was defined as documented VF rhythm from standard 12‐lead ECG or Holter monitoring, or as defined in each study. Sustained ventricular tachycardia was defined as a sustained ventricular rhythm, documented from standard 12‐lead ECG or Holter monitoring, faster than 100 beats per minute lasting at least 30 seconds, or requiring termination earlier because of hemodynamic instability. Only sustained VT, VF, and appropriate defibrillator intervention were included in this study. Nonsustained VT and inappropriate shock were not considered an outcome of interest.

#### 2.4.4 Sudden cardiac death and sudden cardiac arrest

Sudden cardiac death was defined as an unexpected, nontraumatic death that occurred within 60 minutes from the onset of new or worsening symptoms or within 24 hours of last being observed alive.^13^ Sudden cardiac arrest was defined as a sudden cessation of cardiac activity with hemodynamic collapse for which an intervention or spontaneous reversion restores circulation.

### 2.5 Statistical analysis

We performed a meta‐analysis of the included studies using a random‐effects model. Studies were excluded if they did not include an outcome in each intervention group or did not have enough information required for continuous data comparison. We pooled the point estimates of RR from each study using the generic inverse‐variance method of Der Simonian and Laird.^14^ To examine QRS duration, we calculated the weighted mean difference and 95% CIs of QRS duration between BrS patients with and without history of MAE. The heterogeneity of effect size estimates across these studies was quantified using the *I* ^2^ statistic. The *I* ^2^ statistic ranges in value from 0% to 100% (*I* ^2^ < 25%, low heterogeneity; *I* ^2^ = 25%‐50%, moderate heterogeneity; and *I* ^2^ > 50%, substantial heterogeneity).^15^ Publication bias was assessed using a funnel plot and the Egger's regression test^16^ (*P* < .05 was considered significant). All data analyses were performed using the STATA SE version 14.2.

### 2.6 Sensitivity analysis

A sensitivity analysis was performed to assess the influence of the individual studies on the overall results by omitting one study at a time. We used a sequential exclusion strategy, as described by Patsopoulos et al, to examine whether overall estimates were influenced by the substantial heterogeneity observed 17. We sequentially and cumulatively excluded studies that accounted for the largest share of heterogeneity until *I* ^2^ was less than 50%. We then examined whether RR estimates were consistent.

## 3 RESULT

### 3.1 Search results

**Figure 1:**
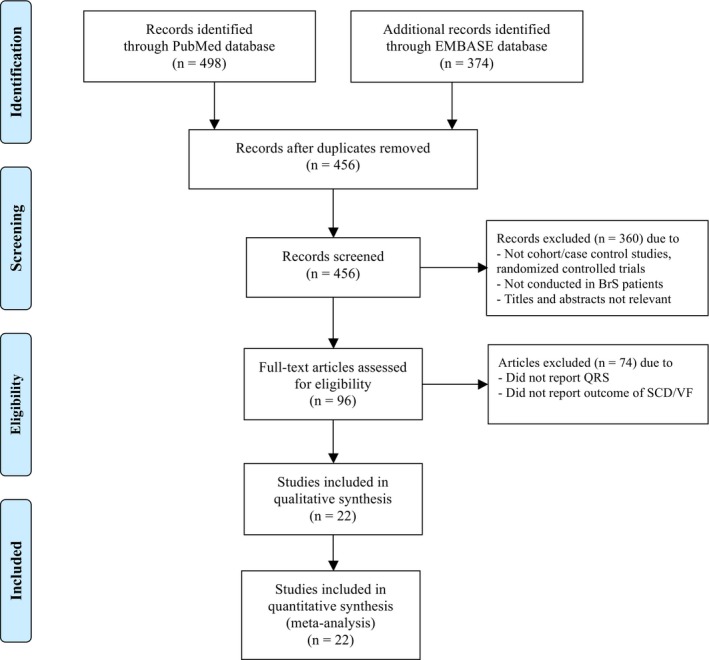
Search methodology and selection process

Our search strategy yielded 872 potentially relevant articles (498 articles from EMBASE and 374 articles from MEDLINE). After the exclusion of duplicated articles, 456 articles underwent title and abstract review. At this stage, 360 articles were excluded as they were not cohort, case‐control, or randomized controlled trials, were not conducted in BrS patients, or the titles and abstracts were not relevant. This left 96 articles for full‐length review. A further 74 studies were excluded as they did not report data regarding QRS duration, reported QRS duration as continuous data, or did not provide sufficient data to calculate HR, RR, or OR. Therefore, a total of 22 studies were included in this meta‐analysis. Figure 1 outlines the search and literature review process.

### 3.2 Description of included studies

A total of 22 studies from 2007 to 2018^6^, ^7^, ^9^, ^18^, ^19^, ^20^, ^21^, ^22^, ^23^, ^24^, ^25^, ^26^, ^27^, ^28^, ^29^, ^30^, ^31^, ^32^, ^33^, ^34^, ^35^, ^36^, ^37^ were included in our meta‐analysis involving 4,814 BrS patients. The mean age was 46.1 ± 12.8 years and patients were predominately men (77.6%) and Caucasian (67.1%). A summary of study characteristics is shown in Table 1. Among 22 included studies, 16 studies^6^, ^18^, ^19^, ^21^, ^22^, ^24^, ^26^, ^28^, ^29^, ^30^, ^31^, ^32^, ^33^, ^34^, ^35^, ^36^ reported mean QRS duration compared between BrS with and without history of MAE and 7 studies^7^, ^9^, ^18^, ^20^, ^23^, ^25^, ^27^ reported the incidence, odds ratio, hazard ratio, or RR of MAE during follow‐up period compared between BrS with wide (>120 milliseconds) and normal QRS duration (≤120 milliseconds).

**Table 1:** Summary characteristics of individual included studies of patients with a Brugada syndrome

Study (year)	Study design	N	Country	Men (%)	Mean age (years)	Symptomatic BrS (%)	Follow‐up (months)	Factors adjusted in analyses	Leads measured	Outcomes
de Asmundis et al, (2017)	Cohort	289	Belgium	70.2	44.8 ± 16.0	35.6	120.6 ± 55.7	N/A	V1‐V3	SCD or appropriate shcok
Benito et al (2008)	Cohort	384	Spain, Belgium, and Canada	70.8	45.9 ± 15.3	21.6	57.9 ± 48.8	N/A	V2	VF or SCD
Calò et al (2016)	Prospective cohort	347	Italy	78.4	45.0 ± 13.1	20.5	48.0 ± 38.6	N/A	V2	VF or SCD
Conte et al (2013)	Retrospective cohort	503	Belgium	58	40.7 ± 12.3	46	29 ± 8	N/A	V1‐V2	sVT or VF
Furushima et al (2005)	Case‐control	24	Japan	95.8	60.0 ± 14.4	62.5	33 ± 16	N/A	V1‐V2	SCA
Ikeda et al (2005)	Prospective cohort	124	Japan	94.4	50.0 ± 15.0	33.1	40.0 ± 19.0	N/A		sVT, VF, or SCD
Junttila et al (2008)	Case‐control	200	Findland, Belgium, China, Spain, and Canada	71.20	40 ± 16	33	N/A	Gender, age, and SCN5A mutation		sVT, VF, or SCD
Kanda et al (2012)	Retrospective cohort	34	Japan	97.1	43.5 ± 12.4	100	38	N/A	V5‐V6	VF or SCA
Kawata et al (2013)	Prospective cohort	49	Japan	94	46.0 ± 12.7	35	93.8 ± 45.6	N/A	N/A	VF or SCD
Kawazoe et al (2016)	Case‐control	143	Japan	97.9	46.17 ± 12.7	32.9	82.8 ± 49.0	N/A	V6	VF
Makarawate et al (2017)	Prospective cohort	40	Thailand	97.5	43.5 ± 12.7	100	28.3 ± 11.3	N/A	N/A	Appropriate shock
Morita et al (2018)	Case‐control	62	Japan	100	50.1 ± 10.9	22.6	27‐134	N/A	V3	VF
Nakano et al (2010)	Prospective cohort	52	Japan	94.2	42 ± 3	34.6	39 + 4	NA	V1‐V2	VF
Nishii et al (2010)	Prospective cohort	108	Japan	97.2	46.8 ± 11.6	38.9	71.9 ± 41.3	N/A	V5	VF
Park et al (2003)		15	Korea	86.7	44 ± 10	87	19 ± 14	N/A	N/A	VF
Probst et al (2010)	Prospective cohort	1029	Italy, Germany, France and The Netherlands	72	45 ± 5	36	40 ± 50	N/A	N/A	SCA
Sieira et al (2017)	Retrospective cohort	400	Belgium	58.3	41.1 ± 17.8	32.7	80.7 ± 57.2	N/A	N/A	SCD
Takagi et al (2007)	Retrospective cohort	188	Japan	94.7	53 ± 14	47.9	37 ± 16	N/A	V6	VF
Take et al (2012)	Retrospective cohort	84	Japan	97.6	47 ± 12	48.8	48 ± 48	N/A	SAEG	VF
Tokioka et al (2014)	Prospective cohort	246	Japan	95.9	47.6 ± 13.6	N/A	45.1 ± 44.3	N/A	V2	SCD, VF
Yamagata et al (2017)	Prospective cohort	415	Japan	97	46 ± 14	45.3	72	N/A	V2	Appropriate shock, SCA or SCD
Zumhagen et al (2016)	Retrospective cros‐section	78	Germany	73.1	45 + 14	65.4	N/A	N/A	V1	sVT, VF, or SCD

Abbreviations: N/A, not applicable; SCA, sudden cardiac arrest; SCD, sudden cardiac death; sVT, sustained ventricular tachycardia; VF, ventricular fibrillation.

#### 3.2.1 Quality assessment of included studies

The NOS of included studies are described in File S2. The NOS uses a star system (0 to 9) to evaluate included studies on 3 domains: selection, comparability, and outcomes. Higher scores represent a higher study quality.

### 3.3 Meta‐analysis results

#### 3.3.1 Wide QRS complex and major arrhythmic event

**Figure 2:**
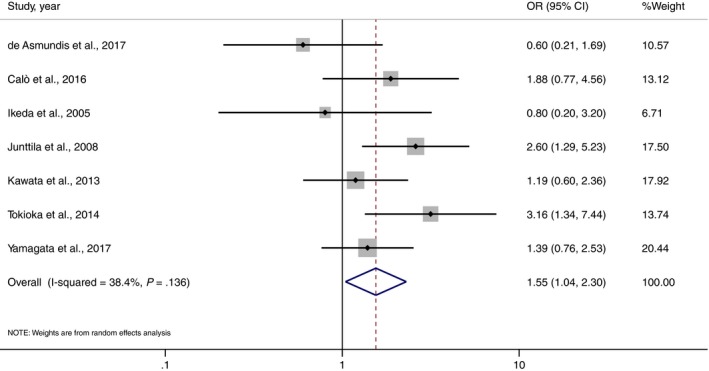
Forest plot demonstrating the association of wide QRS and MAE in patients with Brugada syndrome

**Figure 3:**
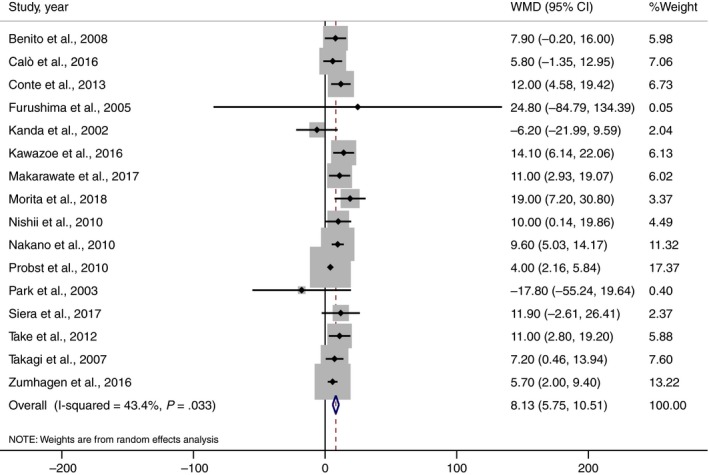
Forest plot demonstrating weight mean difference QRS duration between Brugada syndrome patient with and without history of MAE

Outcomes regarding MAE were available in 7 studies.^7^, ^9^, ^18^, ^20^, ^23^, ^25^, ^27^ There was a significant association between wide QRS complex and an increased risk of MAE (pooled RR = 1.55, 95% CI: 1.04‐2.30, *P* = .03, *I* ^2^ = 38.4%). QRS duration was wider in BrS who had history of MAE (weight mean difference = 8.12 milliseconds, 95% confidence interval: 5.75‐10.51 milliseconds, *I* ^2^ = 43.4%). Forest plot is demonstrated in Figures 2 and 3, respectively.

#### 3.3.2 Sensitivity analysis

To assess the stability of the results of the meta‐analysis, we conducted a sensitivity analysis for each outcome by excluding one study at a time. When omitting Calo et al^18^, Junttika et al^7^, Tokioka et al^9^, or Yamagata et al^20^, wide QRS still increased MAE (RR = 1.49, 1.39, 1.40, and 1.57 respectively) but the results were nonsignificant (File S3).

#### 3.3.3 Publication bias

**Figure 4:**
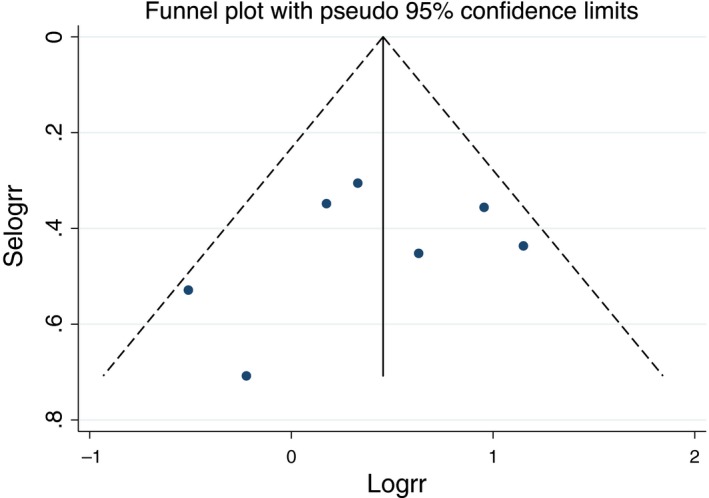
Funnel plot of the association of wide QRS and MAE in patients with Brugada syndrome

**Figure 5:**
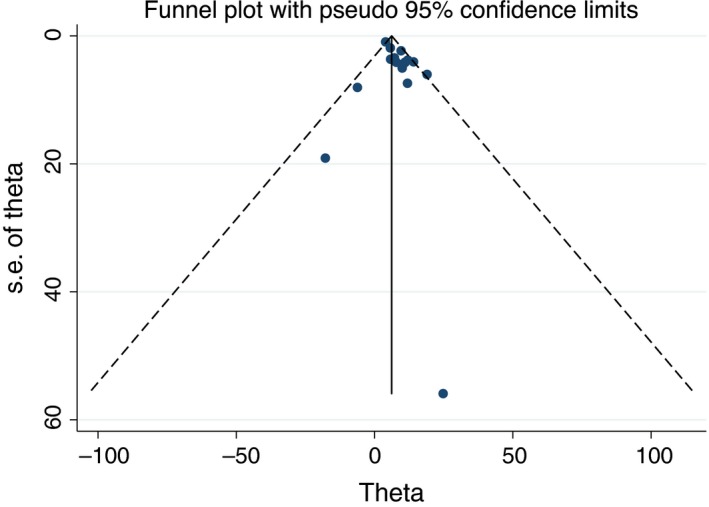
Funnel plot of weight mean difference QRS duration between Brugada syndrome patient with and without history of MAE

We aimed to investigate potential publication bias via funnel plot and Egger's test. No publication bias was observed in Egger's test (*P* = .051 for QRS duration weight mean difference analysis and *P* = .500 for wide QRS analysis) or funnel plot (Figures 4 and 5).^38^, ^39^

## 4 DISCUSSION

The main finding from this meta‐analysis is that the wide QRS complex on surface 12‐lead ECG is associated with an increased risk of MAE in patients with BrS.

Brugada syndrome has been reported to be responsible for up to 20% of SCD in patients with a structurally normal heart, and likely the cause in 4% of all SCD.^40^ Placement of ICD is a class I recommendation in BrS patients with a previous history of MAE, including SCD, SCA, VF, or sustained VT^41^, ^42^ However, a majority of newly diagnosed BrS patients are without previous history of MAE, and thus, they may not meet an indication for ICD placement.^43^ In addition, it remains a challenge to identify asymptomatic patients who are at risk of MAE.^41^

From the current evidence, the only well‐established risk factor for MAE in BrS population is spontaneous type I ECG pattern with a presence of symptoms (previous MAE or syncope presumed to be due ventricular arrhythmias).^42^ This pattern is more commonly found in adult male, Asians ethnicity, and patients with fever.^44^ Other factors that are associated with increased risk of MAE include male gender, proband status, family history of SCD, early repolarization, fragmented QRS, atrial fibrillation, prolonged atrio‐His and His‐ventricular intervals, and inducible ventricular arrhythmia during the electrophysiological study.^6^, ^18^, ^21^, ^45^, ^46^, ^47^, ^48^, ^49^, ^50^, ^51^, ^52^, ^53^, ^54^ To the first of the authors' knowledge, this is the first systematic review and meta‐analysis to evaluate the association between wide QRS complex and risk of MAE in BrS population.

There are three well‐known suggested mechanisms that could explain the ventricular arrhythmia in BrS,^55^ including the repolarization, the depolarization, microscopic fibrosis, myocyte inflammation, and the neural crest models, which are all originated from the right ventricular outflow tract abnormality found in BrS patients.^56^, ^57^, ^58^, ^59^ However, experts believe that there may be other involving factors, yet to be discovered, that also play major role in MAE in BrS patients. Mutation of the SCN5A gene, which encodes the pore‐forming region of the cardiac sodium channel, is the most common gene mutation found in BrS patients, with the impaired sodium channels predisposing patients to phase 2 reentry precipitating ventricular arrhythmias.^6^, ^47^ However, this mutation was only found in less than 30% of the patients with BrS^55^, ^60^ and only shown to be associated with MAE in Asian populations but not Caucasian populations.^20^, ^50^ Most patients with BrS do not have an identifiable mutation. This indicates that there are likely still other causal genes that are yet to be found or other factors not yet described.

A wide QRS complex is a previously described feature shown in the surface ECG of BrS pattern. This conduction abnormality is the likely result of the decreased function of the sodium channel and the abnormal sodium current influx found in an individual with SCN5A gene mutation, leading to lethal arrhythmias in BrS.^4^, ^61^ Ohkubo et al reported that QRS duration >120 milliseconds in lead V2 can be used as a predictor of ventricular arrhythmias in BrS population.^5^ Smits et al found that BrS patients with the SCN5A mutation showed a trend toward longer QRS duration in lead V2, with prolongation of both atrio‐His and His‐Ventricular (HV) interval, compared with the BrS patients without SCN5A mutation.^62^ In addition, Bordachar et al also revealed that patients with an HV interval >55 milliseconds had significantly more atrial arrhythmias than those with a normal HV interval, which subsequently predispose such patients to MAE. The etiology is unclear but could be from L‐type calcium channel mutation or microscopic fibrosis.

Nevertheless, it is important to note that BrS is a heterogeneous disease, meaning that the mechanism of MAE could differ in each patient. Wide QRS is not the only depolarization ECG marker for the increased risk of MAE, as fragmented QRS, QRS dispersion, and presence of late potentials have also been described.^52^, ^63^ Tse et al reported higher QRS dispersion in Type‐1 BrS than non‐Type‐1 BrS. However, no MAE was compared or reported.^64^ Our group published a comprehensive systemic review and meta‐analysis on fragmented QRS as a predictor of arrhythmic event in BrS and showed that baseline fragmented QRS increased major arrhythmic events up to 3‐fold.^52^ Repolarization markers, such as early repolarization and prolonged QT interval, have also been associated with MAE risk.^9^, ^65^, ^66^, ^67^ While a wide QRS complex may have a direct effect to MAE development in certain patients, in other patients this finding is just a sign of a more advanced disease.

### 4.1 Limitations

Our study is not without limitations. We observed moderate heterogeneity (*I* ^2^ = 38.4 and 43.4%) likely because of different patient characteristics, univariate analysis, and measurement leads. The unadjusted studies may be influenced by other confounders causing instability of the result observed in sensitivity analysis. Lastly, despite the association between wide QRS complex and MAE in BrS population, causality cannot be concluded from our study.

## 5 CONCLUSION

Our systematic review and meta‐analysis demonstrate that the presence of wide QRS complex on the 12‐lead ECG is associated with an increased risk of MAE in patients with BrS. This marker can be easily obtained on the surface ECG and can be used to help stratify the risk for MAE in this population.

## CONFLICT OF INTEREST

The authors declare no conflict of interests for this article.
