# Supplementary material for: Wide QRS complex and the risk of major arrhythmic events in Brugada syndrome patients: A systematic review and meta‐analysis
Source: J Arrhythm. 2019 Dec 27;36(1):143–52. doi: 10.1002/joa3.12290 (PMC7011812; doi:10.1002/joa3.12290)
Supplement: Supplementary file 1 [file JOA3-36-143-s001.docx]

MEDLINE

("electrocardiography"[MeSH Terms] OR "electrocardiography"[All Fields] OR "ecg"[All Fields]) AND ("IEEE Int Conf Softw Qual Reliab Secur"[Journal] OR "qrs"[All Fields]) AND brugada[All Fields]

EMBASE

“QRS AND ECG AND brugada AND ([article]/lim OR [article in press]/lim)”
