# Supplementary material for: Wide QRS complex and the risk of major arrhythmic events in Brugada syndrome patients: A systematic review and meta‐analysis
Source: J Arrhythm. 2019 Dec 27;36(1):143–52. doi: 10.1002/joa3.12290 (PMC7011812; doi:10.1002/joa3.12290)
Supplement: Supplementary file 2 [file JOA3-36-143-s002.docx]

Supplement document 2: Newcastle-Ottawa quality assessment scale of included studies in meta-analysis

| First author, publication year | Selection | | | | Comparability | Outcome | | | Total |
| --- | --- | --- | --- | --- | --- | --- | --- | --- | --- |
|  | Represen-tative of exposed cohort | Selection of the non-exposed cohort | Ascertain-ment of exposure | Endpointnot present at start | Comparability (Confounding) | Assessment of outcome | Follow-up duration | Adequacy follow-up |  |
| de Asmundis et al., 2017 | * | * | * | * | * | * | * | * | 9 |
| Benito et al., 2008 | * | * | * | * | * | * | * | * | 8 |
| Calò et al., 2016 | * | * | * | * | ** | * | * | * | 9 |
| Conte et al., 2013 | * | * | * | * | ** | * | * | * | 9 |
| Furushima et al., 2005 | * | * | * | * | * | * | * | * | 8 |
| Ikeda et al., 2005 | * | * | * | * | ** | * | * | * | 9 |
| Junttila et al., 2008 | * | * | * | * | * | * | * | * | 8 |
| Kanda et al., 2012 | * | * | * | * | * | * | * | * | 8 |
| Kawata et al., 2013 | * | * | * | * | * | * | * | * | 8 |
| Kawazoe et al., 2016 | * | * | * | * | ** | * | * | * | 9 |
| Makarawate et al., 2017 | * | * | * | * | ** | * | * | * | 9 |
| Morita et al., 2018 | * | * | * | * | ** | * | * | * | 9 |
| Nakano et al., 2010 | * | * | * | * | ** | * | * | * | 9 |
| Nishii et al., 2010 | * | * | * | * | ** | * | * | * | 9 |
| Park et al., 2003 | * | * | * | * | ** | * | * | * | 9 |
| Probst et al., 2010 | * | * | * | * | ** | * | * | * | 9 |
| Sieira et al., 2017 | * | * | * | * | ** | * | * | * | 9 |
| Takagi et al., 2007 | * | * | * | * | * | * | * | * | 8 |
| Take et al., 2012 | * | * | * | * | ** | * | * | * | 9 |
| Tokioka et al., 2014 | * | * | * | * | ** | * | * | * | 9 |
| Yamagata et al., 2017 | * | * | * | * | ** | * | * | * | 9 |
| Zumhagen et al., 2016 | * | * | * | * | ** | * |  | * | 8 |

Notes: The Newcastle-Ottawa scale uses a star system (0 to 9) to evaluate included studies on 3 domains: selection, comparability, and outcomes. Star (*)= item presents. Maximum 1 star (*) for selection and outcome components and 2 stars (**) for comparability components. Higher scores represent higher study quality.
