# Supplementary material for: Wide QRS complex and the risk of major arrhythmic events in Brugada syndrome patients: A systematic review and meta‐analysis
Source: J Arrhythm. 2019 Dec 27;36(1):143–52. doi: 10.1002/joa3.12290 (PMC7011812; doi:10.1002/joa3.12290)
Supplement: Supplementary file 3 [file JOA3-36-143-s003.pdf]

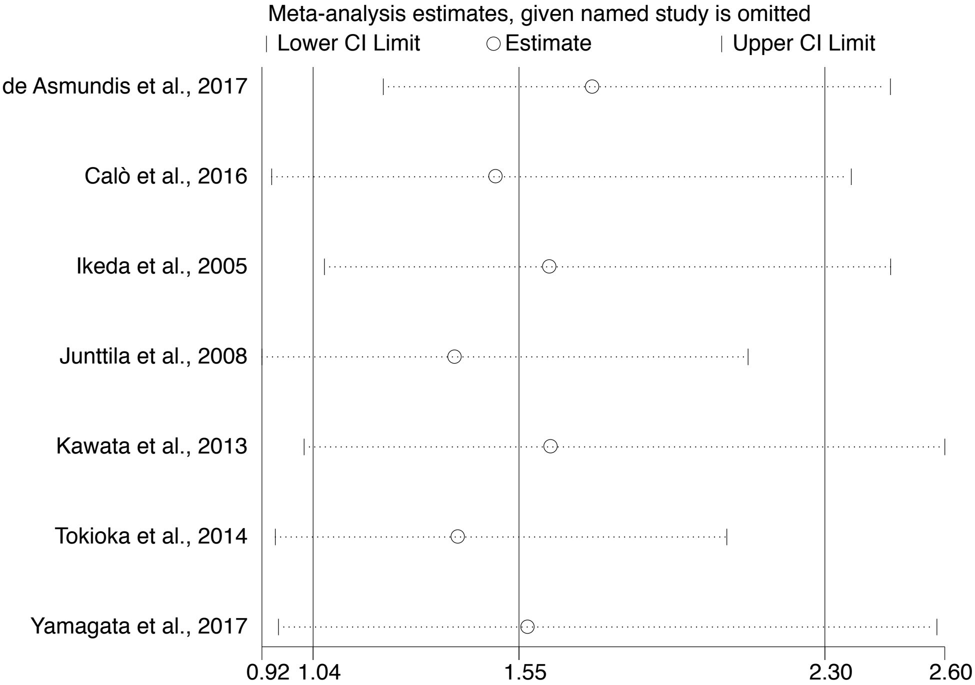

Meta-analysis estimates, given named study is omitted
Lower CI Limit
Estimate
Upper CI Limit
de Asmundis et al., 2017
Calò et al., 2016
Ikeda et al., 2005
Junttila et al., 2008
Kawata et al., 2013
Tokioka et al., 2014
Yamagata et al., 2017
0.92
1.04
1.55
2.30
2.60
